# Call to include breastfeeding as a synergistic approach to vaccines for prevention of respiratory syncytial virus disease

**DOI:** 10.1186/s13006-025-00705-9

**Published:** 2025-03-03

**Authors:** Laura Fischer, Elochukwu Okanmelu, Melissa Ann Theurich

**Affiliations:** 1 Chair of Public Health and Health Services Research, Institute for Biostatistics, Epidemiology, Medical Information Processing, Medical Faculty, Ludwig-Maximilians-Universität München (LMU Munich), Munich, Germany; 2 Pettenkofer School of Public Health, Munich, Germany

**Keywords:** Breastfeeding, Immunization, Vaccine, Lower respiratory infections, Respiratory syncytial virus, RSV, Prevention, Supportive care, Public health communication

## Abstract

**Background:**

Infections with respiratory syncytial virus (RSV) in infancy and early childhood are very common. RSV is the leading cause of bronchiolitis and pneumonia and substantially contributes to the morbidity and mortality of infants and young children worldwide. Until very recently, there have been no vaccines available for prevention and there are no curative treatments for RSV. Two novel pharmaceutical approaches for RSV prevention became available in 2024 namely immunization of mothers during pregnancy and immunoprophylaxis of infants. Since then, a series of scientific papers as well as national and international guidance have been published to encourage parents to vaccinate themselves or their children. Despite strong evidence that breastfeeding is an important non-pharmacological approach for prevention of severe RSV outcomes, recent scientific papers and public health communications have neglected breastfeeding as a core RSV-preventive strategy. This commentary highlights epidemiological evidence of the protective effects of breastfeeding as a key non-pharmacological intervention, discussing its synergistic role in RSV prevention and supportive role in the care of sick infants.

**Breastfeeding and RSV:**

Breastfeeding has been shown to reduce the rate and severity of RSV-associated outcomes, including hospitalization and mortality. While exclusive breastfeeding is most protective, even one month of breastfeeding was associated with a reduced likelihood of Intensive Care Unit admission and the need of mechanical ventilation in RSV-infected infants. The benefits of breastfeeding for RSV prevention and supportive care have been demonstrated in epidemiological studies in low-, middle- and high-income settings and are especially important for small, premature and sick infants.

**Conclusion:**

Breastfeeding is an overlooked sustainable strategy for the universal prevention of severe outcomes and serves as supportive care of RSV-associated disease in infancy, especially in vulnerable population groups. Breastfeeding should be encouraged alongside vaccines in all public health communication, by health providers during pre- and postnatal immunization visits and during infant check-ups. Further, the role of breastfeeding as supportive care of RSV-infected and critically-ill infants should not be overlooked.

## Background

Infection with respiratory syncytial virus (RSV) during infancy and early childhood is very common, with almost all infants and children having been infected by the age of two years [1]. RSV is the leading cause of acute lower respiratory infections (ALRI) such as bronchiolitis and pneumonia in children younger than five years of age. Globally, an estimated 33 million episodes of RSV-associated ALRI were recorded in 2019 and an estimated 101,400 deaths were attributable to RSV; equivalent to one in every 50 deaths in this age group [2]. The burden of RSV is particularly high in infants aged one to six months of age, accounting for an estimated one in every 28 deaths, and in low- and middle-income countries (LMIC) where more than 95% of RSV-associated ALRIs and more than 97% of RSV-attributable deaths occur [2].

RSV is a leading cause of hospitalization and responsible for 3.6 million hospital admissions every year, approximately 39% of which occur in infants aged 0–6 months [2]. Premature and low-birth-weight infants are especially vulnerable to RSV and in this group, lower respiratory infections are one of the three leading causes of disability-adjusted life years (DALYs) [3]. These infants have a higher risk for Intensive Care Unit (ICU) hospitalization from RSV infections, often requiring mechanical ventilation [4–6]. Additional risk factors for severe RSV outcomes include low birth weight, infants with chronic lung disease of prematurity and congenital heart disease [7].

### Treatment of RSV

Supportive care for RSV-infected infants and young children largely focuses on alleviation of symptoms. Minor symptoms (e.g., runny nose, congestion, coughing, sneezing, low-grade fever) are managed with over-the-counter medications. More severe RSV symptoms (e.g., high fever, lethargy, poor feeding, wheezing, difficulty breathing, hypoxia and cyanosis) are managed in-hospital, including intensive care for critically-ill infants (e.g., medical ventilation and supplemental oxygen) [8]. There are no specific curative treatments for RSV. Antiviral agents, such as ribavirin, are sometimes recommended in severe cases, and in immuno-compromised patients [8–10].

### Efficacy of RSV vaccines for prevention of severe outcomes

Until very recently, there had been no available vaccines for RSV prevention [11]. Recent pharmaceutical advances have led to the availability and global recommendation of two vaccines for RSV prevention, namely maternal RSV immunization and the administration of long-acting RSV monoclonal antibodies administered directly to the infant. RSV monoclonal antibodies for infants (nirsevimab) were reported to show efficacy against very severe lower respiratory tract infections (LRTI) from RSV (78.6%, 95% CI 48.8, 91.0) and hospitalization due to LRTI (76.8%, 95% CI 49.4, 89.4) [12]. A maternal RSV vaccine (bivalent RSVpreF maternal vaccine) administered during pregnancy prevented LRTI from RSV (51.3%, 95% CI 29.4, 66.8) and severe LRTI from RSV (69.4%, 95% CI 44.3, 84.1) until six months after birth [12].

### Breastfeeding has long been considered an infant’s first vaccine

Breastfeeding has long been considered an infant’s first vaccine against a myriad of infectious diseases. It has been estimated that breastfeeding could prevent 800,000 deaths of children under the age of five, each year [13]. It strengthens the infant's gut microbiome and immune system, providing numerous immunobiological components including antiviral, antibacterial, and anti-inflammatory properties that protect against respiratory infections, including RSV [14, 15]. This makes breastfeeding technically the most specific personalized medicine for infants with health benefits that should not be missed [13].

### Breastfeeding prevents severe outcomes from RSV and other respiratory infections

The protective effects of breastfeeding on respiratory health are well described in literature, including consolidated evidence on the risks of suboptimal breastfeeding practices for ALRI incidence, hospitalization, and mortality for infants in low-, middle- and high-income countries. A systematic review by Mineva et al. showed that breastfeeding reduces the severity of RSV bronchiolitis, length of hospital stays, and the need for supplemental oxygen [16]. No breastfeeding or only a short duration of breastfeeding (< 2 months) were shown to be significant risk factors for RSV-related hospitalization [16]. A meta-analysis by Lamberti et al. demonstrated the effects of different levels of breastfeeding exposure among infants and children in LMIC [17].

Compared to exclusive breastfeeding, no breastfeeding (RR 5.61; 95% CI 1.23, 25.53) and partial breastfeeding (RR 5.45; 95% CI 1.35, 21.97) increased the relative risk of pneumonia prevalence in the first five months of age [17]. Non-breastfed infants had an increased risk of pneumonia mortality compared to partially-breastfed infants (RR 9.47; 95% CI 2.85, 31.47), and partially-breastfed infants had an increased risk compared to exclusively-breastfed infants (RR 2.50; 95% CI 1.03, 6.04) [17]. A prospective study from Spain added to these findings showing that exclusive breastfeeding reduced incidence of bronchiolitis by 41% (adjusted Incidence Ratio (aIR) 0.59; 95% CI 0.46, 0.76) and mixed breastfeeding reduced incidence by 37% (aIR 0.63; 95% CI 0.47, 0.86) at four months of age [18]. This demonstrates a dose–response relationship between breastfeeding and severe respiratory infection outcomes and underlines the importance of exclusive breastfeeding during the first six months of life.

Furthermore, cohort studies from high-income countries demonstrated protective effects on the severity of RSV-associated infections with even a minimum duration of breastfeeding. Infants who were breastfed the first 14 days of life had significantly lower odds (OR) 0.23; 95% CI 0.07, 0.80) for RSV-associated hospitalization [19]. Just 15 − 28 days of breastfeeding were associated with a reduced likelihood of ICU admission and lower likelihood for needing mechanical ventilation [5]. In a Korean study, RSV-infected hospitalized infants that were formula-fed had increased odds for oxygen therapy compared to exclusively breast milk fed infants (adjusted OR 3.81; 95% CI 1.22, 11.90), suggesting less airway damage in the breastfed group [20]. For these reasons, current clinical guidelines present a strong consensus for the promotion of breastfeeding for preventing severe RSV outcomes in high-risk infants [21].

### Support for the breastfeeding dyad and families during RSV disease

**Fig. 1:**
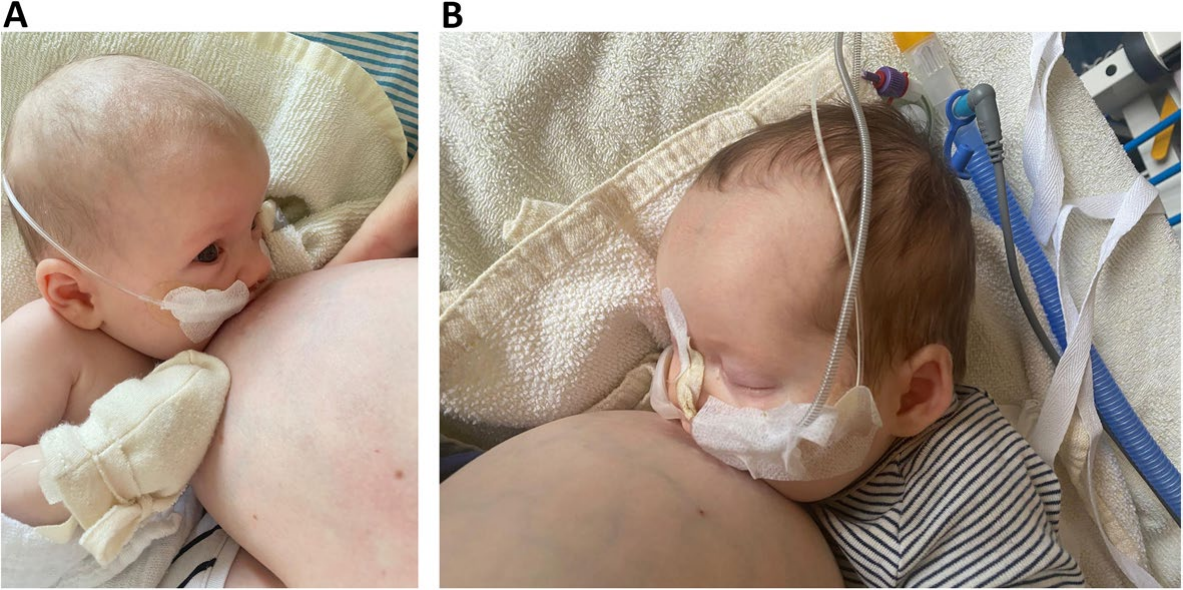
**A** and **B** Exclusively breastfed twins directly breastfeeding while hospitalized with severe RSV infection Legend: Full-term, exclusively breastfed twin infants receiving supportive care in hospital for 2-weeks for severe RSV infection at 5-weeks of age. Both infants required supplemental high-flow oxygen, intravenous fluids and feeding tubes, receiving expressed breast milk through their feeding tubes as well as direct breastfeeding

Sickness and hospitalization pose barriers to breastfeeding [22]. Breast milk feeding should not be considered replaceable, and infants and young children who are breastfeeding should be admitted to hospitals preferably together with their lactating mothers [23, 24]. Health professionals involved in the care of hospitalized infants should enquire about breastfeeding status and not separate RSV-infected infants from their lactating mothers. Breastfeeding dyads should be supported with rooming-in and encouraged to continue breastfeeding throughout the course of the hospital stay [22]. Acutely ill infants can continue breastfeeding at the breast, even while receiving high-flow oxygen and with nasogastric feeding tubes (see Fig. 1A and B). In cases of severe illness when infants are too ill to directly breastfeed, mothers can express their breast milk and feed it using other modalities (e.g., bottle, syringe or feeding tube). Commercial infant formula supplementation is not recommended on the basis of RSV-infection alone and may increase the risk for severe RSV outcomes compared to exclusive breast milk feeding [16].

Lactating mothers should drink adequate fluids to prevent dehydration during illness and breastfeed frequently to avoid milk stasis and related breastfeeding difficulties. Mothers who experience a temporary drop in milk supply should be provided appropriate lactation support. Health facilities’ policies should allow flexibility with visiting family members to support with infant care when lactating mothers are themselves infected with RSV.

RSV vaccination is recommended for grandparents aged 75 and older and adults aged 60–74 who are at increased risk of severe RSV disease progression [25]. Siblings aged 8–19 months with increased medical risk for severe outcomes should also be offered vaccination [25].

### Communication on RSV prevention should include breastfeeding

Current public health communication surrounding the two novel RSV vaccines seem to recommend vaccines in isolation and overlook the synergistic potential of non-pharmaceutical approaches. Modifiable behavioral risk factors for RSV include poor respiratory hygiene, suboptimal breastfeeding practices, maternal smoking during pregnancy or lactation, exposure to passive smoke and other air quality factors [6, 19, 26].

*The Lancet* and *The Lancet Respiratory Medicine* published a series of papers in September 2024 highlighting two novel pharmaceutical approaches for RSV prevention [11, 27]. While briefly discussing hygiene approaches (handwashing, facial masks) and socioeconomic risk factors, the series did not mention breastfeeding as an important protective factor against RSV disease. While the World Health Organization widely promotes breastfeeding, their communication toolkit for RSV disease prevention also focuses solely on vaccines [28]. The United States Center for Disease Control and Prevention [29] provides an overview of options for infant RSV prevention, but does not mention breastfeeding or other modifiable behaviors for the prevention of severe RSV disease [29]. The Robert Koch-Institute shared important considerations entering RSV-season with the German public and mentions behavioral strategies for RSV disease prevention, but overlooks breastfeeding as an important protective factor [30]. The German Federal Centre for Health Education (*Bundeszentrale für gesundheitliche Aufklaerung*, BZgA) provides information for parents on RSV prophylaxis to protect against severe RSV-related respiratory diseases in newborns and infants, but does not include breastfeeding amongst the recommendations [31].

Calls for consistently linking infant immunization and breastfeeding promotion, and communicating breastfeeding as a synergistic approach to vaccines, is not new [32, 33]. In the face of the recent advances in RSV immunization and raised attention for RSV prevention, this commentary highlights the protective effects of breastfeeding for RSV disease prevention and its key role in the supportive care of sick infants. In order to make the most out of efforts to reduce the global burden of RSV disease, health communication to stakeholders and the public around the novel RSV vaccines should include breastfeeding as an essential complement to vaccination. In primary care, breastfeeding should be encouraged alongside vaccines by health providers during pre- and postnatal immunization visits and infant check-ups [34].

## Conclusions

Based on this epidemiological evidence, low global breastfeeding rates and suboptimal breastfeeding practices are potentially hindering wider efforts to reduce the burden of RSV. Despite the body of scientific evidence of the synergistic role of breastfeeding in preventing and managing RSV infections, breastfeeding appears not to be mentioned in many public health communications. This commentary should not be misconstrued to convey that breastfeeding is an equivalent substitution for vaccines or that vaccines are unnecessary. We highlight breastfeeding and human milk as a synergistic approach to vaccination and articulate its supportive role in the treatment of RSV-infected infants. Breastfeeding should be promoted alongside the emerging novel pharmaceutical RSV prevention strategies, for example, during maternal pre- and postnatal immunization visits and infant check-ups. Health providers should provide evidence-based support for breastfeeding during these contacts. Finally, the role of breast milk as supportive care of RSV-infected or critically-ill infants should not be overlooked. Breastfeeding is a highly feasible and effective strategy for prevention of severe RSV outcomes in all countries, especially in LMIC where RSV incidence is highest, survival rates are poor and there is limited access to novel vaccines and pediatric intensive care.

## Data Availability

No datasets were generated or analysed during the current study.

## Abbreviations

ALRI: Acute Lower Respiratory Infection
ICU: Intensive Care Unit
LMIC: Low- and Middle-Income Countries
LRTI: Lower Respiratory Tract Infections
RSV: Respiratory Syncytial Virus
